# Tick-Borne Encephalitis Virus RNA Found in Frozen Goat’s Milk in a Family Outbreak

**DOI:** 10.3390/ijms231911632

**Published:** 2022-10-01

**Authors:** Eirini Mylonaki, Michael Seiberl, Neil Jones, Heike Bernhard, Ferdinand Otto, Georg Pilz, Eugen Trinka, Peter Wipfler

**Affiliations:** 1 Department of Neurology, Christian Doppler University Hospital, Paracelsus Medical University, Centre of Cognitive Neuroscience, 5020 Salzburg, Austria; 2 Department of Paediatrics, Paracelsus Medical University, 5020 Salzburg, Austria; 3 Neuroscience Institute, Christian Doppler University Hospital, 5020 Salzburg, Austria; 4 Department of Public Health, Health Services Research and Health Technology Assessment, UMIT—University for Health Sciences, Medical Informatics and Technology, 05223 Hall in Tirol, Austria

**Keywords:** TBE outbreaks, alimentary infection, food-borne transmission, milk-borne infections, tick-borne encephalitis virus

## Abstract

Tick-borne encephalitis (TBE) is one of the commonest arthropod-borne viral diseases in Middle-East Europe and North Asia. The main reservoir of the virus is comprised of small rodents and domestic mammals with the common tick (Ixodes) being the usual vector. The clinical spectrum of TBE ranges from mild meningitis to severe meningoencephalomyelitis. This disease can lead to severe sequelae and has a mortality up to 2% in Europe. Even though the majority of cases are transmitted through bites of infected ticks, infections through ingestion of contaminated milk and dairy products from farms in endemic areas have been reported. We report a family outbreak of a febrile disease, initially suggestive of human-to-human infection, during the early summertime in Austria. Tick-borne encephalitis was diagnosed following consumption of unpasteurised goat’s milk and the virus was subsequently detected in frozen milk samples. Although this is a rare manifestation of TBE, this case series shows that TBE should be included in the differential diagnosis of an outbreak of febrile disease, and a careful clinical history with reference to unpasteurized dairy products is crucial in order to prevent further disease spread. The best preventive measure is active immunisation of people living in, or travelling to, endemic areas.

## 1. Introduction

Tick-borne encephalitis (TBE) is the most common viral tick-borne zoonosis in Europe and North Asia, causing major morbidity in one third of affected patients [1,2,3]. TBE is caused by an RNA virus of the genus flavivirus, and transmission is primarily through infected ticks. 0.1–5% of ticks are carriers of the tick-borne encephalitis virus (TBEV) [4]. The viral reservoir is comprised of small rodents, migratory birds and domestic mammals [1,5]. The risk of TBE is related to occupational exposure, outdoor leisure activities and travelling from non-endemic to endemic regions [2].

Infection with tick-borne encephalitis virus (TBEV) is usually asymptomatic but can cause serious central nervous system (CNS) inflammation. Mortality rates range from 0.5–2% [1,3,6,7,8,9], and up to 50% of patients suffer long-term neurological complications [1,2].

The diagnosis is supported by a positive history of living in, or travelling to, an endemic area. In central Europe, two peaks in the activity of ticks have been observed in April/May and in September/October [10], thus the majority of cases arise from April to November [1,10]. A cerebrospinal fluid (CSF) pleocytosis and positive TBEV IgM and IgG antibodies in serum, as well as increased antibody index, are diagnostic [3]. There is no effective antiviral treatment for TBE; however, patients often require symptomatic treatment and sometimes even intensive care. Since immunization is effective in preventing TBE [1,3,6,7,8,9], disease morbidity and mortality could be reduced by improving awareness of the disease and instigating vaccination in endemic areas with appropriate recommendations for travellers [9].

## 2. Case Series-Report

The index patient was a 39-year-old man, who was admitted to the Christian Doppler University Hospital Salzburg during the summertime with a 4–5-day history of occipital headache, high fever and photophobia, with preceding malaise and dizziness for 1–2 days. Apart from a temperature of 39.9 °C, clinical examination was normal; in particular, the patient was fully alert and oriented, with no nuchal rigidity or focal neurological signs. There were no other symptoms indicative of a specific infection. The patient had a negative travel history and had no recollection of any tick bites. The medical history was unremarkable, apart from an appendectomy at an age of 24 years.

His 14-year-old son and 41-year-old wife were also admitted to our institution three and eight days later, respectively. The son was suffering from headache, fever of 39.4 °C rigors and nausea over the preceding 3 days, and his wife from headache, non-responsive to over-the-counter medication for 2 weeks and fever up to 39 °C during the last 3 days. At presentation they were both subfebrile (37.6 °C) with a normal neurological examination. This series of febrile illnesses led to the initial suspicion of a human-to-human contagious disease.

**Figure 1:**
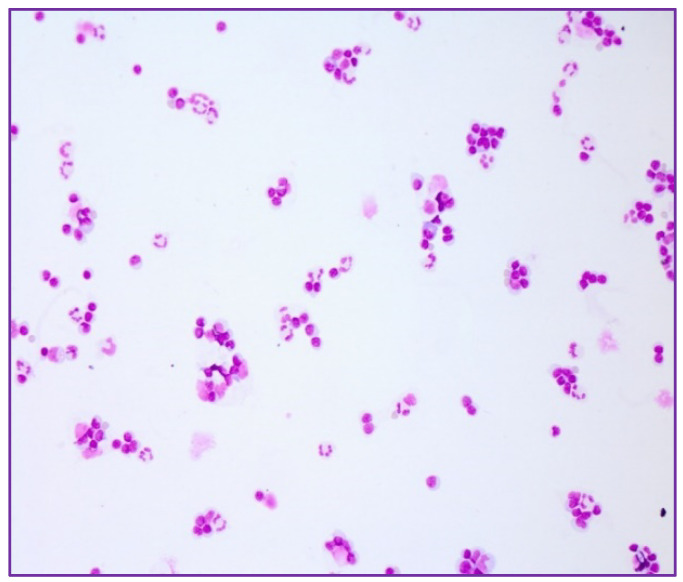
Mild mixed pleocytosis with leukocytes, monocytes and some neutrophils in the CSF of the index patient (20× magnification).

Laboratory results of the index patient showed slight leucocytosis (11.09 G/L), with mainly neutrophils, a reduced number of lymphocytes and no eosinophilia. C-reactive protein (CRP) was within normal limits, and other tests revealed slightly elevated lactate dehydrogenase (LDH) of 346 U/L. Since these cases occurred during the COVID-19 pandemic, SARS-CoV-2 testing was performed, which was negative. The laboratory results of the wife revealed a slight elevated CRP (3.98 mg/dL) and neutrophilic leucocytosis (16.49 G/L) as well as reduced lymphocytes. The blood values of the son were within normal range. The neurological status of the index patient aroused suspicion of meningitis, so a lumbar puncture was performed. This revealed a mixed pleocytosis with 32 cells/μL (Figure 1), with a raised protein (96 mg/dL), borderline lactate (2.4 mmol/L), intrathecal IgM synthesis and brain-blood barrier disturbance. Intravenous acyclovir (750 mg 3 times daily) and ceftriaxone (2 g daily) were started empirically. Serological studies for Borrelia were negative, as well as PCR for herpes viruses (HSV, VSV) and Listeria. Serologically, both blood and CSF samples showed positive TBEV IgM antibodies and borderline TBEV IgG antibodies, which confirmed the diagnosis of TBE. Acyclovir and ceftriaxone were discontinued.

**Figure 2:**
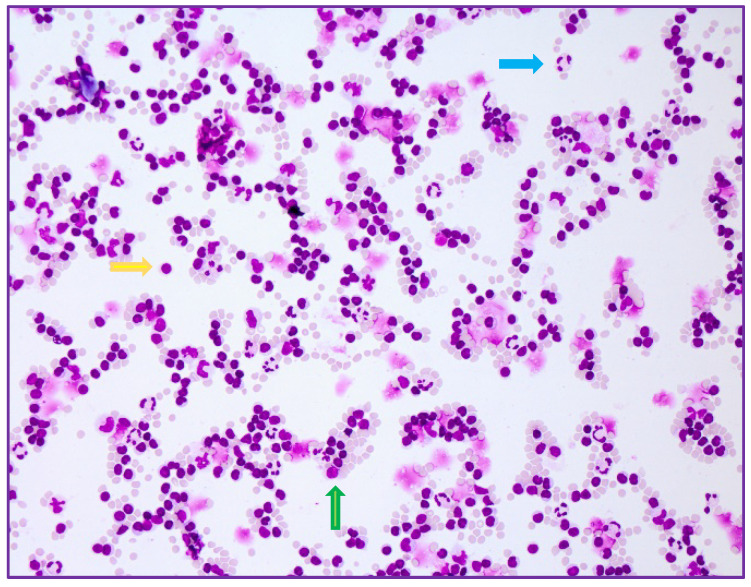
Mixed cell reaction with lymphocytes (yellow arrow), monocytes (green arrow) and neutrophils (blue arrow) in the CSF sample of the 14-year-old patient (20× magnification).

**Figure 3:**
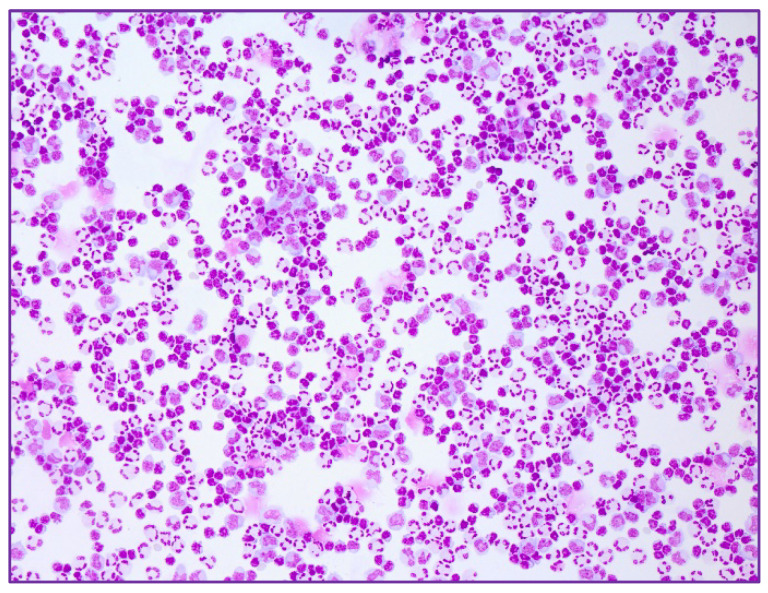
More exacerbated pleocytosis (lymphocytes, monocytes, neutrophils) in the CSF of the 41-year-oldwoman (20× magnification).

The wife and their son also had positive TBEV IgM and borderline IgG antibodies in serum. The lumbar puncture of the son revealed a cell count of 89 cells/μL (Figure 2), with a predominance of lymphocytes, slightly elevated protein (58 mg/dL) and disturbance of the blood-brain barrier. The CSF of the wife showed a mixed but primarily lymphomonocytic pleocytosis of 130 cells/μL (Figure 3), with mild disturbance of blood-brain barrier. No intrathecal antibodies synthesis and no TBEV IgM or IgG antibodies in CSF were found. The samples of all patients were also serologically tested (ELISA) by the reference laboratory of the Centre for Virology of the Medical University of Vienna, and the diagnoses were confirmed.

**Figure 4:**
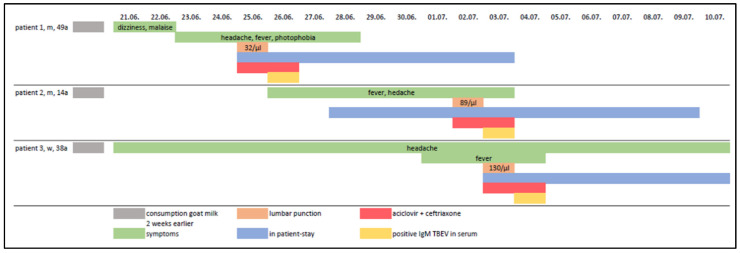
Overview of symptoms, duration of stay, diagnostic results and treatment.

The family could not recall any history of tick bites but, after detailed questioning, it was ascertained that the whole family had been consuming unpasteurised goat’s milk from a farm in the Region of Braunau in Upper Austria, two weeks prior to the onset of symptoms. Based on the history of consumption of raw milk and the absence of a noticed tick bite, a possible alimentary transmission was suspected. The family members were treated symptomatically and after 8 to 12 days of admission they were discharged with resolving headaches and no fever (Figure 4).

Neither the index patient nor his wife had received TBE vaccination. The 14-year-old son had been vaccinated three times, but not according to the standard recommendation (i.e., interval of 12 and 24 months between the first and second and the second and third dose, respectively). The fourth family member, an asymptomatic and TBE unvaccinated 7-year-old boy, had also consumed the same goat’s milk but was negative for TBEV IgM and IgG antibodies in serum.

A container of the frozen goat’s milk at the family home was subsequently tested by the national public health authority laboratory (AGES, the Federal office for Safety in Health Care in Austria), which demonstrated TBEV RNA by quantitative reverse transcriptase polymerase chain reaction (RT-qPCR) and subsequent PCR product sequencing.

## 3. Discussion

We report a family outbreak of tick-borne encephalitis, initially suggestive of human to-human infection, resulting from alimentary transmission.

Due to climate change, TBE is causing increasing concern in Europe [1,9,10]. Over the last 30 years the endemic regions in Europe have expanded, covering many countries in central, northern and eastern Europe, with isolated cases reported from Sicily [11], the Netherlands [12] and Norway [13,14]. The number of reported human cases in Europe has increased by almost 400% in the last 30 years according to data of the European Centre for Disease Prevention and Control (ECDC; https://www.ecdc.europa.eu/en (accessed on 9 August 2022)) [10].

In the majority of cases, the virus is transmitted through bites from TBEV-infected ticks, and alimentary transmission of TBEV, as in our cases, is unusual. Outbreaks due to oral virus transmission have been reported in Eastern Europe and the Baltic states, but have emerged less often in Central Europe [1,3,15,16]. The seroprevalence in domestic animals has been confirmed to correlate with the incidence of TBEV in humans [16]. This food-borne transmission is not considered epidemiologically important, as it accounts for only 1% of cases [1,15,16]. It is however possible that this kind of transmission could be underestimated since data from Germany report that 30–50% of TBE-patients did not recall having a tick bite, which could imply a possible alimentary transmission in some of them [17].

In animals, the virus is rarely pathogenic, and most animals with TBEV-antibodies have no clinical symptoms. Nevertheless, cases with neurological disease in animals such as sheep, goats, horses, monkeys, deer and even dogs have been published [18,19,20,21,22,23,24]. The virulence of virus in milk or dairy products can last up until 25 days [15], depending on storage temperature and the viral load [25].

In our case series, TBE occurred after consumption of unpasteurised goat’s milk. Not all forms of thermal processing of milk or cheese can assure the elimination of the TBEV, but pasteurization and the addition of salt during cheese production is regarded as safe [15,25,26]. The TBE incubation period in milk-borne infection may range from 2 days to 4 weeks [27], while other data suggest a shorter period of 3–7 days [15,17]. The virus survives the acid gastric environment before entering the circulatory system through the duodenum, spreading through lymphatic organs to the blood and then crossing the blood-brain barrier [9,15].

Alimentary infections can be monophasic or biphasic, with an initial flu-like phase (3–7 days) and a second phase with high temperature, meningitis or encephalitis [15]. The symptoms in severe cases usually resolve after 12–21 days but sometimes over several months. The course of the disease and symptoms of alimentary infection may vary [15]. In our case-series, the index patient and his wife both had a biphasic course, their 14 year old son had a rather monophasic one (Figure 4), whilst the other 7-year-old boy remained uninfected. In another case-series of food-born outbreak, there was a 50% prevalence of monophasic disease [28].

According to surveillance data of the ECDC, in year 2019, 98.3% of all TBE cases (of those with a known immunisation status) had not been vaccinated [29]. In our case-series, the index patient and his wife were unvaccinated. The 14-year-old son had received three doses of the vaccine, but not according to the recommended immunisation schedule [30], and we believe that this breakthrough infection could be attributed to the prolonged delay between his first and second vaccination doses. Ineffective vaccine response due to delay between doses has been reported [30]. In another case-series of alimentary transmission, none of the patients had been vaccinated [28].

## 4. Conclusions

In TBE endemic regions physicians should be aware that a cluster of fever and headaches within a family during the end of spring till autumn could be secondary to alimentary transmission of TBE. Health education of people living in endemic areas, especially farmers and consumers of dairy products, could help in the prevention of TBEV [15,17]. Food-borne infection can be eliminated by pasteurization of milk products [1,2,9,15].

In addition, the risk of TBE can be reduced by using insect repellents and protective clothing to prevent tick bites [9]. Full vaccination against TBEV, according to the recommended schedule [30], is the most effective prophylaxis against TBE caused by both tick-borne as well as food-borne infections [1,9,15]. Appropriate campaigns to raise vaccine awareness should be initiated.

Lastly, while tourism is increasing, TBE should be included in the differential diagnosis of CNS infections in those returning from endemic regions, emphasizing the importance of obtaining a travel history [1]. It should be kept in mind that even without a tick bite, an alimentary transmission after traveling in endemic areas is possible.

## Data Availability

Not applicable.
